# Cerebral Small Vessel Disease Burden in Acute Ischemic Stroke and the Role of Physical Activity: Cross‐Sectional Study

**DOI:** 10.1002/brb3.71165

**Published:** 2026-01-18

**Authors:** Andreas Gammelgaard Damsbo, Rolf Ankerlund Blauenfeldt, Sigrid Breinholt Vestergaard, Niels Lech Pedersen, Kim Morgenstjerne Ørskov, Mette Foldager Hindsholm, Arzu Bilgin‐Freiert, Claus Ziegler Simonsen, Søren Paaske Johnsen, Rikke Beese Dalby, Grethe Andersen, Janne Kaergaard Mortensen

**Affiliations:** ^1^ Danish Stroke Centre, Department of Neurology Aarhus University Hospital Aarhus Denmark; ^2^ Faculty of Health, Department of Clinical Medicine Aarhus University Hospital Aarhus Denmark; ^3^ Steno Diabetes Center Aarhus Aarhus University Hospital Aarhus Denmark; ^4^ Department of Radiology Aarhus University Hospital Aarhus Denmark; ^5^ Department of Neurosurgery Aarhus University Hospital Aarhus Denmark; ^6^ Danish Center for Health Services Research, Department of Clinical Medicine Aalborg University Aalborg Denmark; ^7^ Department of Radiology and Nuclear Medicine University Hospital of Southern Denmark Esbjerg Denmark

**Keywords:** cerebral small vessel disease, ischemic stroke, physical activity

## Abstract

**Background:**

Cerebral small vessel disease (cSVD) is a major cause of stroke and cognitive decline. While classical cardiovascular risk factors are well‐established contributors to overall cSVD burden, the effect of physical activity (PA) is not fully understood. This study aims to investigate the association between PA and cSVD in patients with acute ischemic stroke (AIS).

**Methods:**

This is a post hoc analysis of data from two randomized stroke trials. cSVD burden was quantified on acute admission magnetic resonance imaging (MRI) markers (microbleeds, lacunes, white matter hyperintensities, and atrophy) with scores ranging 0–4. Pre‐stroke PA was assessed by admission questionnaire and categorized into quartiles (first quartile is lowest PA level). Association of PA and cSVD burden was analyzed using ordinal logistic regression.

**Results:**

A total of 762 patients with AIS were included. The median (IQR) age was 71 (62, 79), and 279 (37 %) were females. Patients with a cSVD score of 0 constitutes 26%, 38%, 43%, and 57%, through the first to fourth PA quartile. Analyses adjusting for age and sex of higher cSVD score showed the odds ratios of 0.64 (confidence intervals: 0.44–0.93) in the second PA quartile, 0.79 (0.53–1.16) in the third, and 0.51 (0.33–0.76) in the fourth quartile compared to the first and lowest quartile. Multivariable analysis showed 0.63 (0.43–0.93) in second, 0.86 (0.57–1.29) in third, and 0.56 (0.36–0.87) in fourth quartile compared to the first quartile, adjusting for sex, age, lifestyle factors, cardiovascular disease, and pre‐stroke functional impairment.

**Conclusion:**

Among patients with AIS, we found a statistically significant association between the highest PA quartile and lower cSVD burden. The direction of causality cannot be determined due to the study design, but warrants further testing in randomized trials.

## Graphical abstract

In total, 762 patients with acute ischemic stroke, available acute clinical magnetic resonance imaging, and data on physical activity prior to stroke were included, with cSVD features consisting of cerebral microbleeds, lacunes, white matter hyperintensity, and global cerebral atrophy.

Higher physical activity level was found to be associated with lower cerebral small vessel disease burden, and among the different cSVD features, the association was strongest for atrophy.

A potential causality may be bidirectional, and further studies are needed.

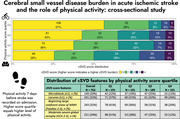

## 1 Introduction

Cerebral small vessel disease (cSVD) is one of the main causes of stroke, while also an important contributor to the risk of dementia (Adams et al. 1993; Livingston et al. 2024).

The cSVD is a multifactorial disease entity comprised of several underlying pathologic processes affecting the global cerebral circulation (Duering et al. 2023). Several features on magnetic resonance imaging (MRI) have been identified as biomarkers of cSVD, including cerebral microbleeds, chronic lacunes of presumed vascular origin, deep white matter hyperintensity (WMH), and atrophy, which have been recommended as measures of the overall cSVD burden (Duering et al. 2023; Markus et al. 2022).

Physical activity (PA) is appreciated as an important element in preventing and reducing cardiovascular risk factors such as hypertension and diabetes, and reducing the negative effect of smoking (Whelton et al. 2002; Dylewicz et al. 1999), all risk factors shared by cSVD, stroke, and dementia. Guidelines on stroke and dementia prevention include PA (Livingston et al. 2024; Kleindorfer et al. 2021; Wardlaw et al. 2024), but the association between cSVD and PA is still not clear (Buchman et al. 2018; Landman et al. 2021; Anderson et al. 2024).

The assumed effect of PA on cSVD is multifaceted. PA may reduce classical cardiovascular risk factors mentioned previously, modify the effect of these cardiovascular risk factors, and may act directly on cerebral vessels by improving endothelial function and lowering circulating fibrinogen levels (Ernst 1993; Sherman 2000; Hamakawa et al. 2013; Boa Sorte Silva et al. 2022; Maleki et al. 2022; Chen et al. 2025).

A higher level of PA has been associated with lower cSVD burden in the general population and among patients with cognitive impairment (Anderson et al. 2024; Boa Sorte Silva et al. 2022; Del Brutto et al. 2020; Moniruzzaman et al. 2020), while studies describing this association among patients with stroke are scarce.

We aimed to investigate how PA correlates to the burden of cSVD among patients with acute ischemic stroke (AIS) by evaluating the distribution of cSVD burden across different levels of pre‐stroke PA, adjusted for common cardiovascular risk factors.

## 2 Methods

### 2.1 Study Population

This study is a post‐hoc, cross‐sectional study on baseline MRI data from patients with AIS, pooled from two large randomized clinical trials: the Efficacy of Citalopram Treatment in Acute Ischemic Stroke (TALOS) and the Remote Ischemic Conditioning for Acute Stroke (RESIST) trials (Kraglund et al. 2018; Blauenfeldt et al. 2023).

The TALOS trial included patients with first‐time stroke within 7 days after stroke onset who were randomized to citalopram or placebo treatment (Kraglund et al. 2018). In the RESIST trial, patients presenting with a prehospital putative acute stroke within 4 h of symptom onset were randomized in the ambulance to ischemic conditioning treatment or sham treatment (Blauenfeldt et al. 2023).

Patients included in this current study were all admitted to the comprehensive stroke center at Aarhus University Hospital, Aarhus, Denmark, between 2013 and 2022. All received a final diagnosis of AIS and had a pre‐stroke PA assessment available as well as an acute MRI scan. The acute MRI protocol conducted included diffusion weighted imaging (DWI) sequence, a fluid attenuated inversion recovery (FLAIR) sequence, as well as T2*‐weighted or susceptibility‐weighted imaging (SWI) sequences.

The current study is approved by the local ethics committee. Data were collected in accordance with the principles of the Declaration of Helsinki.

### 2.2 CSVD Annotation and Scoring

Based on the available MRI sequences, cSVD biomarker annotation was evaluated for each subject by two independent, trained assessors according to the Standards for Reporting Vascular Changes on Neuroimaging (STRIVE) definitions (Wardlaw et al. 2013). In case of disagreement, consensus scoring was performed by a third independent assessor. Assessors were blinded to clinical patient data and other assessors’ evaluations.

MRI biomarkers of cSVD have been combined in different ways for overall scoring systems of the cSVD burden in prognostication of long‐term outcome (Huijts et al. 2013; Olama et al. 2020) and cSVD progression (Landman et al. 2021).

**FIGURE 1:**
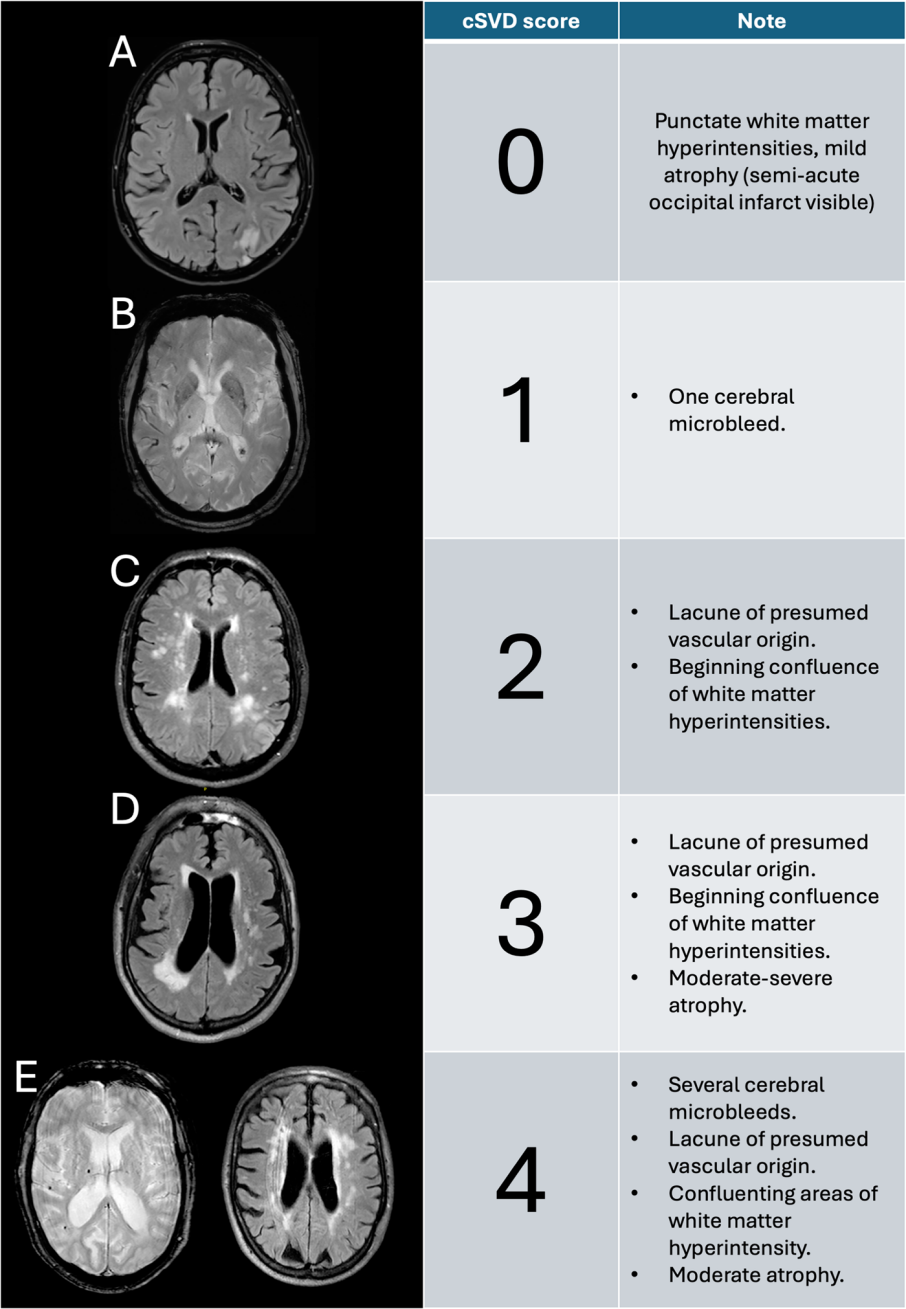
Example scores of the simplified cSVD score used from different subjects. Panel B and E (left) are from T2*‐weighted sequences, the other panels are all FLAIR sequences.

In this study, the cSVD score was calculated by allocating one point for presence of each of the following: cerebral microbleeds (≥ 1), lacunes of presumed vascular origin (≥ 1), white matter hyperintensity (Fazekas score 2–3 in deep white matter) and global cerebral atrophy (Global Cortical Atrophy score 2–3) (Klarenbeek et al. 2013; Henneman et al. 2009). Thus, the cSVD score had a range of 0–4 with a higher score indicating more severe cSVD burden. Cerebral microbleeds were evaluated on T2* or SWI sequences, while the other cSVD markers were evaluated based on their presence on the FLAIR sequence (Figure 1).

All MRI scans were performed on scanners with a magnetic field strength of 1.5 or 3 Tesla.

### 2.3 Clinical Data

All clinical data were retrieved from the Danish Stroke Registry (Mainz 2004). The registry includes demographic and lifestyle data and we included data on age and sex as well as smoking habits (never, former, active), alcohol consumption (above 7 or 14 units of alcohol weekly for females and males, respectively), cohabitation status (living alone) and pre‐stroke functional status (assessed by the modified Rankin Scale [mRS]), and data on hypertension, diabetes, prior cerebral ischemic event (AIS or transitory ischemic attack [TIA]), previous myocardial infarction (MI) and atrial fibrillation (AFib). Having diabetes, hypertension, or AFib was defined by either receiving treatment, having a current diagnosis at the time of stroke admission, receiving a diagnosis, or starting treatment during the hospital stay. All other characteristics were registered on stroke admission.

### 2.4 Physical Activity

Pre‐stroke PA was defined by the PA level during the past 7 days prior to stroke. Pre‐stroke PA was assessed using the Physical Activity Scale for the Elderly (PASE) questionnaire on trial enrolment (Washburn et al. 1999). PASE is a validated 12‐item questionnaire on overall PA, including a variety of everyday activities, including work, leisure time, household, and sports activities. The different items are summed through a weighted algorithm to give a score ranging from 0 to above 700, with a higher score indicating a higher level of PA. In a cohort of sedentary, older individuals (mean age 66.5), a median PASE score of 123 was found (Washburn et al. 1999). The PASE questionnaire was completed by the patient or next of kin, and if necessary, with the assistance of study personnel.

In the analyses, the PA score is divided into quartiles, with the first quartile corresponding to the lowest PA scores.

### 2.5 Statistics

Baseline characteristics of included patients are presented in a table, and the distribution of cSVD scores stratified by PA level is visualized in stacked bar plots. Comparisons of baseline factors were performed using the Kruskal–Wallis rank sum test and Pearson's Chi‐squared test where relevant. All calculations were performed using 95% confidence intervals (CI).

The correlation of PA level and cSVD burden was evaluated using ordinal logistic regression to evaluate the odds of a higher cSVD burden score with PA level as the main exposure.

In the regression analyses, age and sex were considered confounders of the association between PA and cSVD, while all other available covariables were considered potential mediators. This led to different adjustment strategies for the regression analyses: an unadjusted, univariable model; a minimal, confounder‐adjusted model adjusted for age and sex; and a multivariable model with all available variables included. A directed acyclic graph was used to illustrate this strategy (see Figure S1).

To further explore the association between cSVD and PA, several sensitivity analyses were performed. The regression analyses were repeated on the data stratified by sex as well as on data filtered to only include patients with pre‐stroke mRS of 0 and without previous ischemic events (AIS or TIA). Additionally, to account for the heterogeneity in the MRI sequences used for microbleeds detection, regression analyses were repeated using a short cSVD score excluding the microbleed feature. The quality of this model was compared to the main regression analyses by comparing the Akaike information criterion (AIC) of the confounder‐adjusted models. To assess the individual association of PA and each cSVD feature, logistic regression models were created with adjustment strategies similar to the main analyses. The quality of each of these models was compared based on the AIC.

Cases with incomplete data were excluded from the regression analyses. Due to the small number of missing observations (see Table 1), no data imputation was performed.

**TABLE 1:** Baseline characteristics and cSVD score characteristics stratified by physical activity quartile 1–4 (lowest‐highest).

Characteristic	Overall *N* = 762	Q1 *N* = 191	Q2 *N* = 191	Q3 *N* = 189	Q4 *N* = 191
Age, median (IQR)	71 (62–79)	77 (67–84)	74 (65–80)	70 (63–76)	64 (55–73)
Female sex, *n* (%)	279 (37%)	85 (45%)	89 (47%)	58 (31%)	47 (25%)
Living alone, *n* (%)	203 (27%)	70 (37%)	54 (29%)	45 (24%)	34 (18%)
Missing, *n*	4	1	2	1	0
Smoking, *n* (%)					
Never	269 (36%)	57 (31%)	75 (40%)	69 (38%)	68 (36%)
Current	211 (28%)	56 (31%)	45 (24%)	53 (29%)	57 (30%)
Prior	262 (35%)	70 (38%)	66 (35%)	62 (34%)	64 (34%)
Missing	20CS	8	5	5	2
High alcohol consumption, *n* (%)	69 (9.2%)	19 (10%)	19 (10%)	20 (11%)	11 (5.8%)
Missing, *n*	15	6	4	3	2
Hypertension, *n* (%)	418 (55%)	123 (64%)	114 (60%)	96 (51%)	85 (45%)
Diabetes, *n* (%)	85 (11%)	27 (14%)	27 (14%)	19 (10%)	12 (6.3%)
Previous ischemic event, *n* (%)	97 (13%)	30 (16%)	24 (13%)	27 (14%)	16 (8.4%)
Atrial fibrillation, *n* (%)	116 (15%)	34 (18%)	29 (15%)	29 (15%)	24 (13%)
Previous MI, *n* (%)	58 (7.6%)	11 (5.8%)	13 (6.8%)	19 (10%)	15 (7.9%)
Pre‐stroke mRS, *n* (%)					
0	605 (79%)	121 (63%)	150 (79%)	159 (84%)	175 (92%)
1	89 (12%)	29 (15%)	22 (12%)	23 (12%)	15 (7.9%)
2	61 (8.0%)	35 (18%)	18 (9.4%)	7 (3.7%)	1 (0.5%)
3	7 (0.9%)	6 (3.1%)	1 (0.5%)	0 (0%)	0 (0%)
cSVD score characteristics					
cSVD score, median (IQR)	1 (0–2)	1 (0–2)	1 (0–2)	1 (0–2)	0 (0–1)
cSVD score features					
Microbleeds (≥ 1), *n* (%)	149 (20%)	42 (22%)	37 (19%)	47 (25%)	23 (12%)
Lacunes (≥ 1), *n* (%)	241 (32%)	71 (37%)	60 (31%)	61 (32%)	49 (26%)
Beginning‐large confluent areas of WMH (Fazekas 2–3), *n* (%)	243 (32%)	78 (41%)	69 (36%)	58 (31%)	38 (20%)
Moderate‐severe global atrophy (GCA 2–3), *n* (%)	221 (29%)	98 (51%)	53 (28%)	47 (25%)	23 (12%)

Abbreviations: cSVD, cerebral small vessel disease; GCA, global cortical atrophy; MI, myocardial infarction; mRS, modified Rankin Scale; WMH, white matter hyperintensity.

No multicollinearity was detected in the multivariate regression models evaluated by the variance inflation factor. The proportional odds assumption of the ordinal logistic regression models was tested using the Brant test and was met (Brant 1990).

## 3 Results

In total, 762 patients with available MRI and PA data were included: 397 from the RESIST cohort and 365 from the TALOS trial. Overall, RESIST patients were older, had a lower PASE score, fewer smoked regularly, and more had hypertension and/or previous TIA. The TALOS cohort did not include patients with previous AIS, as these were first‐time stroke patients.

All baseline characteristics are presented in Table 1. Overall, median age was 71 (IQR: 62–79), 279 (37 %) were female, and median PASE score was 108 (IQR: 60–160). Complete data were present on 733 subjects. Patients in the lower PA quartiles were older, and more were females, more had hypertension, diabetes, AFib, and higher pre‐stroke mRS scores compared to the higher quartiles.

**FIGURE 2:**
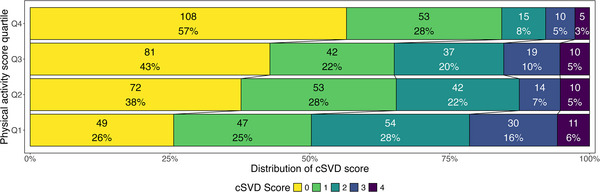
Relative distribution of cerebral small vessel disease (cSVD) burden score stratified by physical activity quartiles (Q1–Q4). The cSVD score considers the presence of microbleeds, presence of lacunes, beginning‐large confluent areas of WMH and/or moderate‐severe global atrophy for a complete score of 0–4. Numbers are absolute numbers and proportion for each score in each group.

Stratified by PA level, patients with a cSVD score of 0 represented 26%, 38%, 43%, and 57% respectively, from the lowest to the highest PA quartile (Figure 2).

### 3.1 Regression Analyses

The ordinal regression models showed that a high PA level was associated with lower odds of a higher cSVD burden (Table 2).

**TABLE 2:** Ordinal regression models of cSVD burden score as the main outcome with PASE score as the main exposure, evaluating the odds ratio of higher cSVD score with a given PASE quartile compared to the first PASE quartile (lowest level of PA).

	Univariable	Minimal	Multivariable
Characteristic	OR (95% CI)	OR (95% CI)	OR (95% CI)
Pre‐stroke PA quartile			
Q1 (lowest PA level)	—	—	—
Q2	0.57 (0.39–0.81)	0.64 (0.44–0.93)	0.63 (0.43–0.93)
Q3	0.52 (0.36–0.75)	0.79 (0.53–1.16)	0.86 (0.57–1.29)
Q4 (highest PA level)	0.26 (0.17–0.37)	0.51 (0.33–0.76)	0.56 (0.36–0.87)

*Note*: A total of 733 subjects with complete data were included in the multivariable analysis. The minimally adjusted model is adjusted for age and sex. The multivariable model is adjusted for all available covariables.

Abbreviations: CI, confidence interval; OR, odds ratio; PA, physical activity.

The odds ratio (OR) for a higher cSVD score was 0.57 (CI: 0.39–0.81) for the second PA quartile, 0.52 (0.36–0.75) for the third, and 0.26 (0.17–0.37) for the fourth (highest) PA quartile, compared to the first (lowest) PA quartile.

When adjusting for age and sex, a high PA level was still associated with lower odds of a higher cSVD burden. In the minimally adjusted model, the ORs (CIs) were 0.64 (0.44–0.93) for the second PA quartile, 0.79 (0.53–1.16) for the third, and 0.51 (0.33–0.76) for the fourth PA quartile, as compared to the first quartile.

When adjusting for all available covariables, the ORs (CI) were 0.63 (0.43–0.93) for the second PA quartile, 0.86 (0.57–1.29) for the third, and 0.56 (0.36–0.87) for the fourth PA quartile compared with the first quartile (Table 2). Results from univariate analyses of all considered covariables and all coefficients from the multivariable analyses are reported in the supplementary material (Table S1 and Figure S2).

Sensitivity analyses found the same overall pattern of correlation between cSVD score and PASE quartiles among males, females, and patients without pre‐stroke functional disability (mRS score 0) or prior ischemic event (AIS or TIA) (Tables S2 and S3).

Evaluating a short cSVD score excluding microbleeds, a similar distribution of cSVD scores across PASE score quartiles were found (Figure S3), and the regression analyses showed the same overall pattern of lower odds of increased cSVD score with higher PA level (Table S4). Compared to the main regression analyses, the model evaluating the short cSVD achieved the lowest AIC.

Performing logistic regression analyses to evaluate the individual impact of each assessed cSVD feature showed that only moderate‐severe global atrophy showed statistically significant lower odds with higher PA quartile (Table S5). Evaluating overall model quality showed that this model on atrophy achieved the lowest AIC.

## 4 Discussion

This was a cross‐sectional study of the association between pre‐stroke PA level and cSVD burden on acute MRI in a large cohort of patients with AIS.

Higher PA levels were associated with a lower cSVD burden, with patients in the highest PA quartile having 74% lower odds of an increased cSVD score. The minimally adjusted regression model adjusting for age and sex changed the point estimates of model coefficients, but the associations remained statistically significant. These results remained consistent, although reduced after further adjustments, indicating that the effect of PA on cSVD is mediated through other pathways than just modifying classical cardiovascular risk factors. Evaluating the association of PA level and each feature of the cSVD score indicates that the global cerebral atrophy may be the main driver behind this association.

While an association between PA and cSVD has been shown previously (Landman et al. 2021; Del Brutto et al. 2020; Moniruzzaman et al. 2020; Anderson et al. 2024), it has not been studied in a stroke population. One study included 590 older community‐dwelling individuals (Del Brutto et al. 2020). Similar to our results, they found an association between lower levels of PA and higher white matter hyperintensity volume as well as a higher number of microbleeds and lacunes. Another study on PA and progression of overall cSVD burden among 503 patients diagnosed with cSVD in a prospective design with a 9‐year follow‐up showed no association between baseline PA and neither overall cSVD progression nor progression on individual cSVD markers (Landman et al. 2021). PA assessment was based on a questionnaire estimating the average time spent on different activities per week during the last year, which may have increased the risk of recall bias and diluted the results. The authors only used baseline PA in their analyses and did not take changes in the PA level into account. The distribution of cSVD burden scores by PA level was also not reported, which limits the comparability to the current study.

Finally, a large study among 680 stroke‐free Japanese males investigated the association of baseline daily step‐counts and cSVD biomarkers after ∼7 years of follow‐up (Moniruzzaman et al. 2020). A moderate level of PA was correlated to the lowest prevalence of cSVD, suggesting that a very high level of PA is not necessarily protective to the cerebral microcirculation, or should be differentiated when addressing neuroprotectivity.

Limitations of this current study include that the diagnoses of comorbid diseases were registered without any data on treatment status or severity, and did not include biomedical measures such as Hemoglobin A1C, lipid profile, or body mass index.

Furthermore, due to the nature of the study design, no clear conclusions on causality can be drawn without risking overlooking reverse causality, as patients suffering from cSVD may have gradually become more inactive before events leading to inclusion in a stroke cohort study. By including patients with stroke only, the cohort represents a group of patients at high cardiovascular risk and not the general population. The relationship between PA and cSVD risk factors is complex, probably bidirectional, and a clear inference of results from an observational or cross‐sectional study like the present will never be conclusive.

We used the PASE questionnaire to assess pre‐stroke PA upon stroke admission, which has been validated to reflect retrospective PA (Washburn et al. 1999). The PASE questionnaire was chosen as it focuses on specific activities, and assessment was performed at a median of 2 days after stroke admission, to help mitigate the risk of recall bias in patients suffering from acute illness. If necessary, study personnel assisted in the completion of the questionnaire, which may have reduced the validity of the results, but also meant we were able to include patients with more severe stroke symptoms.

Only patients with available PASE scores were included, which may have resulted in a selection bias, as patients with more severe strokes or more severe cSVD may have been excluded.

Only 37% of patients included were female, which reduces the generalizability of our findings. This reflects the proportion of females included in the original trials, but not in the general stroke population (Buus et al. 2022). We did, however, repeat our analyses stratified by sex and found no major differences in the results.

The exclusion of patients without a PASE score, as well as the relatively low representation of women, may have added to underestimating the true association between PA and cSVD.

The assessment of cSVD features was based on acute stroke scans performed over a period of almost a decade. All scans were performed at the same comprehensive stroke center, but on different scanners with different field strengths (1.5 or 3 T). The introduction of the SWI sequence in the stroke protocol to replace the T2* sequence also meant that microbleed detection may have varied across subjects. To address this, we repeated the main analyses based on a short cSVD score excluding microbleeds, and thereby only including features assessed on the FLAIR sequence. This model showed a similar association between PA and cSVD but achieved a lower AIC, reflecting a higher model quality.

In this study, we found higher cSVD scores among patients with lower PA levels. The regression analyses reflected these findings, showing a consistent trend towards lower cSVD in higher PA levels, though after applying adjustments, only the association among the highest level of PA remained statistically significant. This may have been caused by an overall low level of PA in this stroke population or the dilution of contrast due to the limitations mentioned above, which would result in only showing a statistically significant lower odds in the highest quartile, the level with the highest contrast.

This large study on 762 patients with AIS investigated the cSVD burden distribution and the association of recent PA level prior to stroke. The regression analyses showed a statistically significant association of higher PA level and a lower cSVD burden. Both confounder‐adjusted and multivariate models were included, indicating that PA is associated with cSVD through other pathways than just the modification of traditional cardiovascular risk factors. This study does not answer the directional nature of the association between cSVD and PA in stroke patients, but highlights the association in a patient population with high cardiovascular risk. Further studies to explore the causality between cSVD and PA are warranted.

## Author Contributions

**Andreas Gammelgaard Damsbo**: conceptualization, investigation, writing – original draft, methodology, validation, visualization, formal analysis, software, project administration, data curation. **Rolf Ankerlund Blauenfeldt**: conceptualization, investigation, funding acquisition, methodology, validation, writing – review and editing, project administration, supervision, data curation. **Sigrid Breinholt Vestergaard**: writing – review and editing, investigation, data curation. **Niels Lech Pedersen**: writing – review and editing, data curation. **Kim Morgenstjerne Ørskov**: writing – review and editing, data curation. **Mette Foldager Hindsholm**: writing – review and editing, data curation. **Arzu Bilgin‐Freiert**: writing – review and editing, data curation. **Claus Ziegler Simonsen**: writing – review and editing, data curation, conceptualization, investigation, methodology. **Søren Paaske Johnsen**: conceptualization, writing – review and editing, supervision, investigation. **Rikke Beese Dalby**: conceptualization, writing – review and editing, methodology, validation, supervision. **Grethe Andersen**: supervision, conceptualization, investigation, funding acquisition, writing – review and editing, methodology, validation, data curation, resources, project administration. **Janne Kaergaard Mortensen**: conceptualization, investigation, writing – review and editing, validation, methodology, supervision, data curation, project administration.

## Funding

The authors have nothing to report.

## Ethics Statement

The TALOS trial was approved by the local institutional review board and the Committees on Health Research Ethics (ID: 1‐10‐72183‐13). The RESIST trial was approved by Danish regional research ethics committees (ID: 1‐10‐72‐97‐17). The current study is approved by the local ethics committee. Data were collected in accordance with the principles of the Declaration of Helsinki. All patients were included by written informed consent, or if necessary, consent from next of kin.

## Conflicts of Interest

The authors declare no conflicts of interest.

## Supporting information



Supplementary Information

## Data Availability

This study is based on sensitive data subject to EU legal restrictions. A subset of the data can, however, be made available from the corresponding author on a reasonable request.
