# Supplementary material for: Cerebral Small Vessel Disease Burden in Acute Ischemic Stroke and the Role of Physical Activity: Cross‐Sectional Study
Source: Brain Behav. 2026 Jan 18;16(1):e71165. doi: 10.1002/brb3.71165 (PMC12813408; doi:10.1002/brb3.71165)
Supplement: Supplementary file 1 — Supplementary Information [file BRB3-16-e71165-s001.docx]

Cerebral small vessel disease burden in acute ischemic stroke and the role of physical activity: cross-sectional study

Short title: Cerebral small vessel disease and physical activity in stroke

**Authors:** Andreas Gammelgaard Damsbo, MD*^1,2,3^, Rolf Ankerlund Blauenfeldt, MD PhD^1,2^, Sigrid Breinholt Vestergaard, MD PhD^1,2^, Niels Lech Pedersen, MD^2,4^, Kim Morgenstjerne Ørskov, MD^1,2^, Mette Foldager Hindsholm, MD^1,2^, Arzu Bilgin-Freiert, MD PhD^2,5^, Claus Ziegler Simonsen, MD PhD^1,2^, Søren Paaske Johnsen, MD PhD^6^, Rikke Beese Dalby, MD PhD^7^, Grethe Andersen, MD DMSc^1,2^, Janne Kaergaard Mortensen, MD PhD^1,2^

**Affiliations**:

1: Danish Stroke Centre, Department of Neurology, Aarhus University Hospital, Aarhus, Denmark

2: Faculty of Health, Department of Clinical Medicine, Aarhus University, Aarhus, Denmark

3: Steno Diabetes Center Aarhus, Aarhus University Hospital, Aarhus, Denmark

4: Department of Radiology, Aarhus University Hospital, Aarhus, Denmark

5: Department of Neurosurgery, Aarhus University Hospital, Aarhus, Denmark

6: Danish Center for Health Services Research, Department of Clinical Medicine, Aalborg University, Aalborg, Denmark

7: University Hospital of Southern Denmark, Department of Radiology and Nuclear Medicine, Esbjerg, Denmark

*Corresponding author: [andrlr@rm.dk](mailto:andrlr@rm.dk), Palle Juel-Jensens Boulevard 165, DK-8200 Aarhus N, Denmark


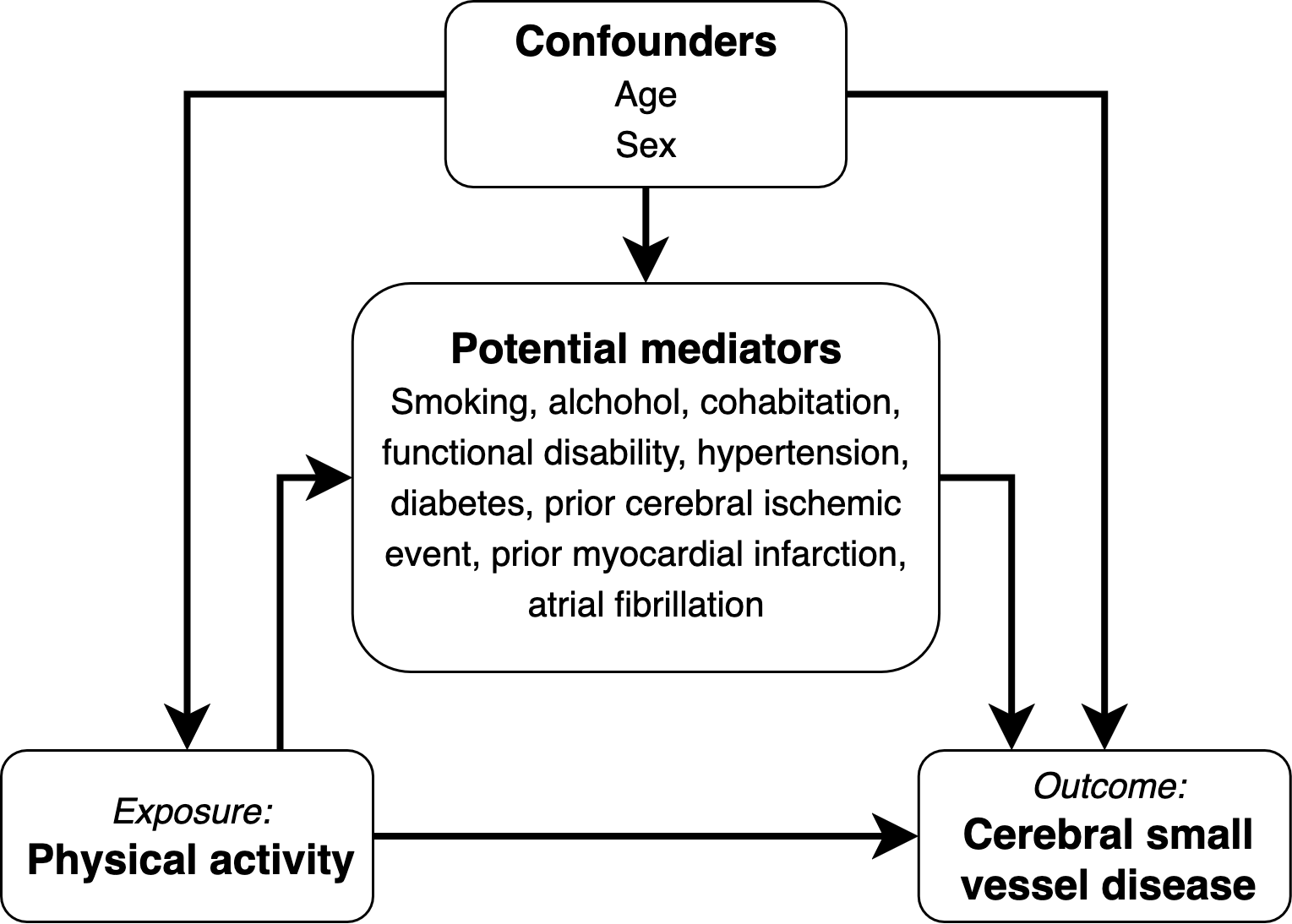


Supplementary figure 1 Simplified directed acyclic graph illustrating the relationship between physical activity, cerebral small vessel disease, confounders and potential mediators.

For simplicity all confounders were depicted in on box, and all potential mediators were depicted in one box. This graph is not exhaustive.

|  | Univariable | | | Multivariable | |
| --- | --- | --- | --- | --- | --- |
| **Characteristic** | **N** | **OR** | **95% CI** | **OR** | **95% CI** |
| Pre-stroke PA quartile | 762 |  |  |  |  |
| Q1 |  | — | — | — | — |
| Q2 |  | 0.57 | 0.39, 0.81 | 0.63 | 0.43, 0.93 |
| Q3 |  | 0.52 | 0.36, 0.75 | 0.86 | 0.57, 1.29 |
| Q4 |  | 0.26 | 0.17, 0.37 | 0.56 | 0.36, 0.87 |
| Age | 762 | 1.09 | 1.08, 1.11 | 1.09 | 1.07, 1.11 |
| Female sex | 762 | 1.19 | 0.91, 1.55 | 0.86 | 0.62, 1.18 |
| Living alone | 758 | 1.56 | 1.17, 2.07 | 1.05 | 0.75, 1.47 |
| Smoking | 742 |  |  |  |  |
| never |  | — | — | — | — |
| current |  | 1.01 | 0.73, 1.41 | 1.51 | 1.04, 2.20 |
| prior |  | 1.69 | 1.24, 2.32 | 1.36 | 0.97, 1.90 |
| High alcohol consumption | 747 | 1.45 | 0.94, 2.24 | 1.32 | 0.83, 2.07 |
| Hypertension | 762 | 2.90 | 2.22, 3.81 | 1.81 | 1.35, 2.45 |
| Diabetes | 762 | 1.50 | 1.01, 2.23 | 1.33 | 0.85, 2.07 |
| Previous ischemic event | 762 | 2.53 | 1.71, 3.75 | 2.06 | 1.36, 3.14 |
| Atrial fibrillation | 762 | 1.05 | 0.73, 1.50 | 0.56 | 0.37, 0.83 |
| Previous MI | 762 | 0.87 | 0.53, 1.42 | 0.52 | 0.30, 0.88 |
| Pre-stroke mRS | 762 |  |  |  |  |
| 0 |  | — | — | — | — |
| 1 |  | 1.58 | 1.06, 2.35 | 1.04 | 0.68, 1.59 |
| 2 |  | 3.06 | 1.90, 4.95 | 0.98 | 0.57, 1.68 |
| 3 |  | 2.97 | 0.92, 9.73 | 0.56 | 0.14, 2.28 |

Supplementary table 1 Univariable and multivariable ordinal regression analyses of higher small vessel disease burden. Includes univariable analyses of all considered covariables and includes all coefficients from the multivariable analyses. The multivariable analysis is the same as the main analysis as seen in Table 2 in the main article.

PA: Physical activity; MI: Myocardial Infarction; mRS: modified Rankin Scale; SVD: small vessel disease


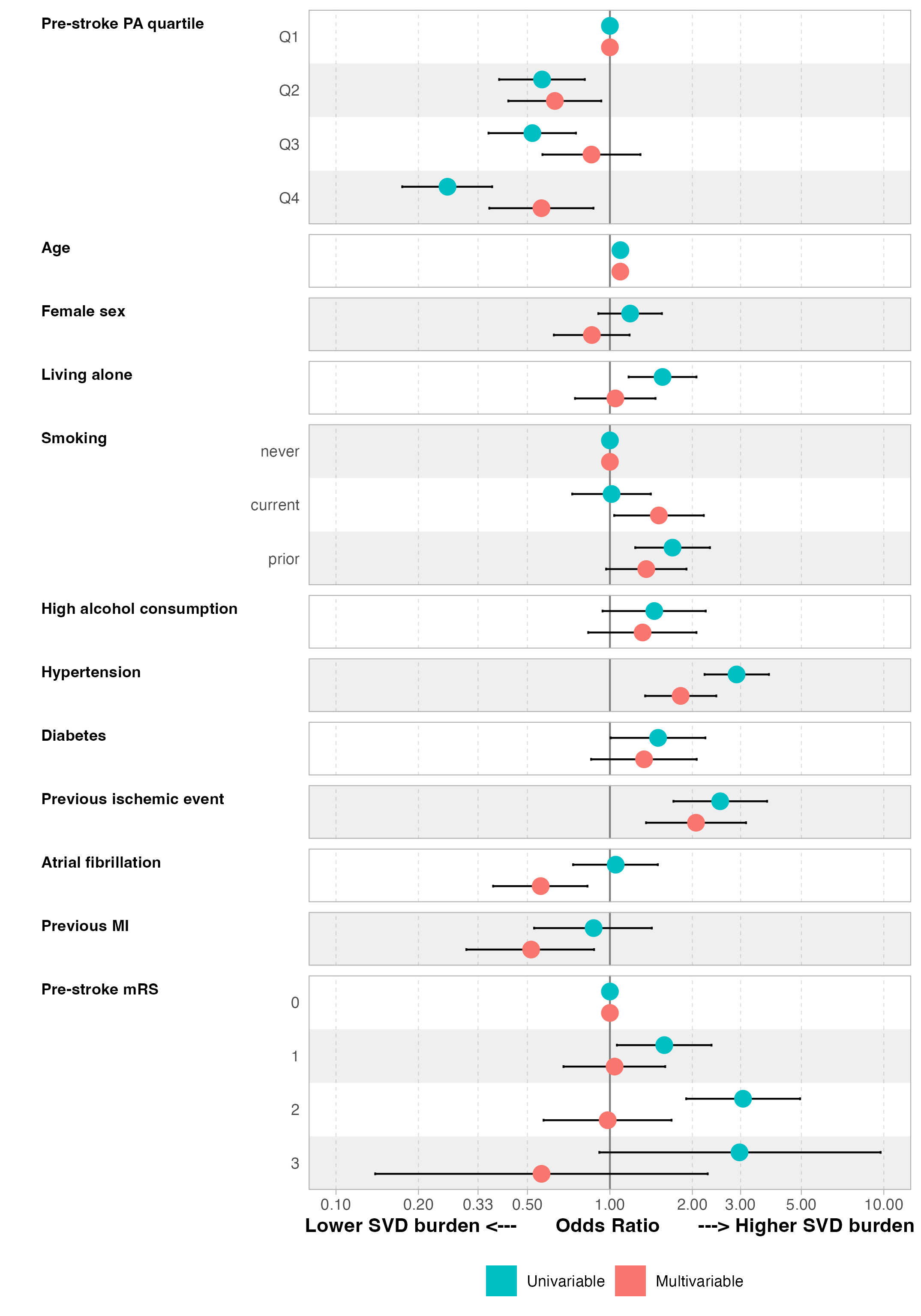


Supplementary figure 2 Univariable and multivariable ordinal regression analyses coefficients of higher small vessel disease burden plotted. Based on the values reported in Supplementary table 1.

PA: Physical activity; MI: Myocardial Infarction; mRS: modified Rankin Scale; SVD: small vessel disease

|  | Univariable | Minimal | | Multivariable | |
| --- | --- | --- | --- | --- | --- |
| **Characteristic** | **OR** **(95% CI)** | **OR** **(95% CI)** | **p-value** | **OR** **(95% CI)** | **p-value** |
| **MALE** | | | | | |
| Pre-stroke PA quartile |  |  |  |  |  |
| Q1 | — | — |  | — |  |
| Q2 | 0.72 (0.44 to 1.17) | 0.73 (0.44 to 1.20) | 0.21 | 0.71 (0.42 to 1.20) | 0.20 |
| Q3 | 0.60 (0.38 to 0.96) | 0.73 (0.45 to 1.19) | 0.21 | 0.86 (0.51 to 1.44) | 0.57 |
| Q4 | 0.31 (0.19 to 0.49) | 0.49 (0.30 to 0.80) | 0.005 | 0.58 (0.34 to 0.97) | 0.040 |
| **FEMALES** | | | | | |
| Pre-stroke PA quartile |  |  |  |  |  |
| Q1 | — | — |  | — |  |
| Q2 | 0.42 (0.24 to 0.72) | 0.56 (0.32 to 0.98) | 0.044 | 0.51 (0.27 to 0.95) | 0.034 |
| Q3 | 0.43 (0.23 to 0.78) | 0.94 (0.48 to 1.84) | 0.86 | 1.02 (0.48 to 2.13) | 0.96 |
| Q4 | 0.19 (0.09 to 0.38) | 0.59 (0.27 to 1.29) | 0.18 | 0.61 (0.26 to 1.43) | 0.26 |

Supplementary table 2 Ordinal regression models of SVD burden score as main outcome stratified by sex with PASE score quartile as the main exposure

CI: Confidence Interval; OR: Odds Ratio; PA: Physical activity

|  | Univariable | Minimal | Multivariable |
| --- | --- | --- | --- |
| **Characteristic** | **OR** **(95% CI)** | **OR** **(95% CI)** | **OR** **(95% CI)** |
| Pre-stroke PA quartile |  |  |  |
| Q1 | — | — | — |
| Q2 | 0.59 (0.37 to 0.93) | 0.62 (0.39 to 1.00) | 0.65 (0.39 to 1.06) |
| Q3 | 0.55 (0.35 to 0.86) | 0.87 (0.54 to 1.41) | 1.03 (0.62 to 1.70) |
| Q4 | 0.30 (0.19 to 0.47) | 0.62 (0.38 to 1.02) | 0.78 (0.46 to 1.32) |

Supplementary table 3 Ordinal regression models of SVD burden score as main outcome with PASE score quartile as the main exposure only including patients with pre-stroke modified Rankin Scale score of 0 (no disability) and no previous ischemic events (acute ischemic stroke or transient ischemic attack).

CI: Confidence Interval; OR: Odds Ratio; PA: Physical activity


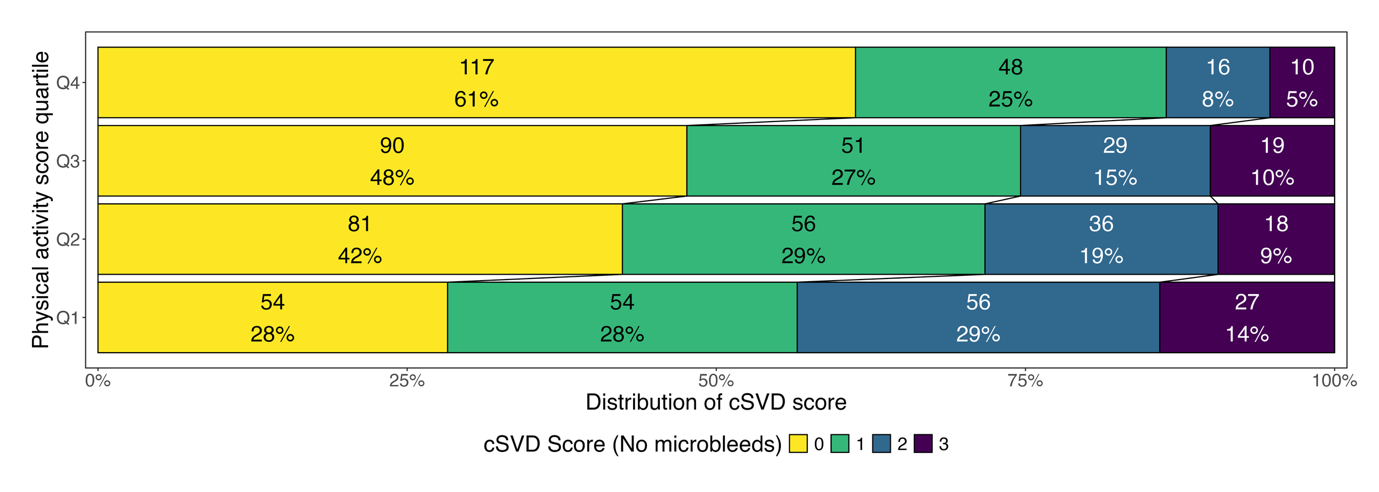


Supplementary figure 3 Relative distribution of a short version of the cerebral small vessel disease (cSVD) burden score stratified by physical activity quartiles (Q1-4).

The short version cSVD score considers the presence of lacunes, beginning-large confluent areas of WMH and/or moderate-severe global atrophy for a complete score of 0-3. Numbers are absolute numbers and proportion for each score in each group.

|  | **Univariable** | **Minimal** | **Multivariable** |
| --- | --- | --- | --- |
| **Characteristic** | **OR (95% CI)** | **OR (95% CI)** | **OR (95% CI)** |
| Pre-stroke PA quartile |  |  |  |
| Q1 | — | — | — |
| Q2 | 0.54 (0.38 to 0.78) | 0.62 (0.42 to 0.91) | 0.62 (0.42 to 0.93) |
| Q3 | 0.46 (0.31 to 0.66) | 0.68 (0.46 to 1.01) | 0.75 (0.50 to 1.15) |
| Q4 | 0.25 (0.17 to 0.36) | 0.49 (0.32 to 0.75) | 0.56 (0.36 to 0.88) |

Supplementary table 4 Ordinal regression models of the short cSVD burden score (0-3) as main outcome with PASE score quartile as the main exposure.

The short version cSVD score considers the presence of lacunes, beginning-large confluent areas of WMH and/or moderate-severe global atrophy for a complete score of 0-3.

CI: Confidence Interval; OR: Odds Ratio; PA: Physical activity; PASE: physical activity scale in the elderly

|  | **Univariate** | **Minimal** | **Multivariate** |
| --- | --- | --- | --- |
| **Characteristic** | **OR (95% CI)** | **OR (95% CI)** | **OR (95% CI)** |
| **Microbleeds (≥ 1)** | | | |
| Pre-stroke PA quartile |  |  |  |
| Q1 | — | — | — |
| Q2 | 0.85 (0.52 to 1.40) | 0.90 (0.55 to 1.49) | 0.81 (0.48 to 1.38) |
| Q3 | 1.17 (0.73 to 1.89) | 1.38 (0.85 to 2.28) | 1.38 (0.82 to 2.34) |
| Q4 | 0.49 (0.28 to 0.84) | 0.64 (0.35 to 1.14) | 0.66 (0.35 to 1.20) |
| **Lacunes (≥ 1)** | | | |
| Pre-stroke PA quartile |  |  |  |
| Q1 | — | — | — |
| Q2 | 0.77 (0.51 to 1.18) | 0.83 (0.54 to 1.28) | 0.80 (0.50 to 1.27) |
| Q3 | 0.81 (0.53 to 1.23) | 0.92 (0.59 to 1.43) | 0.93 (0.58 to 1.50) |
| Q4 | 0.58 (0.38 to 0.90) | 0.74 (0.46 to 1.18) | 0.81 (0.49 to 1.34) |
| **Beginning-large confluent areas of WMH** | | | |
| Pre-stroke PA quartile |  |  |  |
| Q1 | — | — | — |
| Q2 | 0.82 (0.54 to 1.24) | 0.94 (0.60 to 1.49) | 0.97 (0.60 to 1.57) |
| Q3 | 0.64 (0.42 to 0.98) | 1.06 (0.66 to 1.71) | 1.18 (0.71 to 1.95) |
| Q4 | 0.36 (0.23 to 0.57) | 0.80 (0.48 to 1.34) | 0.90 (0.52 to 1.56) |
| **Moderate-severe global atrophy** | | | |
| Pre-stroke PA quartile |  |  |  |
| Q1 | — | — | — |
| Q2 | 0.36 (0.24 to 0.56) | 0.36 (0.22 to 0.60) | 0.36 (0.21 to 0.62) |
| Q3 | 0.31 (0.20 to 0.48) | 0.43 (0.25 to 0.73) | 0.45 (0.25 to 0.80) |
| Q4 | 0.13 (0.08 to 0.22) | 0.22 (0.12 to 0.41) | 0.25 (0.13 to 0.48) |

Supplementary table 5 Logistic regression models of each item in the cSVD score as outcome and pre-stroke PASE score quartile as main exposure.

CI: Confidence Interval; OR: Odds Ratio; PA: Physical activity; PASE: physical activity scale in the elderly
